# Outcomes after laparoscopic cholecystectomy in patients older than 80 years: two-years follow-up

**DOI:** 10.1186/s12893-024-02383-6

**Published:** 2024-03-12

**Authors:** Camilo Ramírez-Giraldo, Luis Carlos Venegas-Sanabria, Susana Rojas-López, Violeta Avendaño-Morales

**Affiliations:** 1 Surgery Department, Hospital Universitario Mayor – Méderi, Bogotá, Colombia; 2 Research Department, Hospital Universitario Mayor – Méderi, Bogotá, Colombia; 3 Universidad del Rosario, Bogotá, Colombia; 4 Grupo de Investigación Clínica, Escuela de Medicina y Ciencias de la Salud, Universidad del Rosario, Bogotá, Colombia

**Keywords:** Elderly, Cholecystitis, Cholecystectomy, Surgery

## Abstract

**Background:**

The laparoscopic cholecystectomy is the treatment of choice for patients with benign biliary disease. It is necessary to evaluate survival after laparoscopic cholecystectomy in patients over 80 years old to determine whether the long-term mortality rate is higher than the reported recurrence rate. If so, this age group could benefit from a more conservative approach, such as antibiotic treatment or cholecystostomy. Therefore, the aim of this study was to evaluate the factors associated with 2 years survival after laparoscopic cholecystectomy in patients over 80 years old.

**Methods:**

We conducted a retrospective observational cohort study. We included all patients over 80 years old who underwent laparoscopic cholecystectomy. Survival analysis was conducted using the Kaplan‒Meier method. Cox regression analysis was implemented to determine potential factors associated with mortality at 24 months.

**Results:**

A total of 144 patients were included in the study, of whom 37 (25.69%) died at the two-year follow-up. Survival curves were compared for different ASA groups, showing a higher proportion of survivors at two years among patients classified as ASA 1–2 at 87.50% compared to ASA 3–4 at 63.75% (*p* = 0.001). An ASA score of 3–4 was identified as a statistically significant factor associated with mortality, indicating a higher risk (HR: 2.71, CI95%:1.20–6.14).

**Conclusions:**

ASA 3–4 patients may benefit from conservative management due to their higher risk of mortality at 2 years and a lower probability of disease recurrence.

## Background

Patients with benign biliary disease, including symptomatic cholelithiasis, biliary origin pancreatitis, gallbladder polyps, choledocholithiasis, and cholecystitis, have conditions that increase with age [1]. When this pathology presents itself, the gold standard for prevention and/or treatment is laparoscopic cholecystectomy [2–4]. However, in older adults, the performance of laparoscopic cholecystectomy results in poorer surgical outcomes, with a higher mortality rate and procedure-related complications. This is more evident in octogenarian and nonagenarian patients [5–8]. Therefore, more conservative alternatives have been evaluated for managing these patients to reduce morbidity and mortality, such as cholecystostomy, endoscopic retrograde cholangiopancreatography (ERCP) [9], or antibiotic treatment alone in cases of cholecystitis [10].

The worst surgical outcomes in older adults are multifactorial and do not depend solely on age, but rather on other factors associated with advanced age. Among these factors are sarcopenia, frailty, malnutrition, and functional dependence [11, 12]. On the other hand, multiple comorbidities and a higher frequency of emergency procedures in this age group have also been linked to worse surgical outcomes [13].

One of the drawbacks of conservative management for patients with benign biliary disease is the recurrence of the disease, which can be as high as 39.8% within the first 2 years [10]. However, in octogenarian and nonagenarian patients, this recurrence may be lower due to the probability of death from other causes in this age group, as they have already surpassed their life expectancy. Life expectancy in our population is 77.87 years, while the global average is 73.16 years for the year 2023 [14].

Given the above, it is necessary to evaluate survival after laparoscopic cholecystectomy in patients over 80 years old to determine whether the long-term mortality rate is higher than the reported recurrence rate. If so, this age group could benefit from a more conservative approach, such as antibiotic treatment or cholecystostomy. Therefore, the aim of this study was to evaluate the factors associated with 2 years survival after laparoscopic cholecystectomy in patients over 80 years old.

## Patients and methods

### Study Design

We conducted a retrospective observational cohort study. Convenience sampling was performed. All patients over 80 years old who met the inclusion criteria were selected. Clinical data was extracted from a database designed for a previous study [15]. Data about death and date of death were extracted from the national database of the Administrator of the Resources of the General System of Social Security in Health (ADRES). This study was reviewed and approved by the Ethics Commit and the Technical Research Commit (approval number DVO005 2402-CV1544). We followed the STROBE guidelines to report this study [16].

### Patients

Patients under 80 years old, those with planned open cholecystectomy, preoperative diagnosis of gallbladder cancer, cholecystectomy combined with another surgical procedure (such as gastrectomy or pancreatoduodenectomy), without postoperative follow-up appointment, and patients whose records lacked the variables of interest were excluded.

The indication for laparoscopic cholecystectomy in all cases was for benign disease (biliary colic, pancreatitis, choledocholithiasis, gallbladder polyps, cholecystitis, or a combination of these). In all cases, at least one image confirming biliary disease was available. In cases of cholecystitis, the severity was diagnosed, classified, and treated according to the Tokyo guidelines [17, 18]. Additionally, we followed the American guidelines for the risk of choledocholithiasis, in which low-risk cases underwent cholecystectomy without additional studies, intermediate-risk cases underwent magnetic resonance cholangiopancreatography (MRCP), and high-risk cases underwent ERCP [19]. In cases of pancreatitis, cholecystectomy was defined when clinically resolved.

All patients had a follow-up outpatient appointment where clinical evolution, surgical wound status, and histopathological results of the surgical specimen were reviewed, along with recording mortality within 2 years following the procedure.

We analyzed the following data: patient demographics, body mass index, ASA Physical Status Classification, presence of diabetes mellitus, hypertension, chronic obstructive pulmonary disease, chronic kidney disease, cardiovascular disease, liver disease, use of anticoagulants or antiplatelet agents, preoperative laboratory results, indication for the surgical procedure, bile duct diameter in preoperative images, severity classification in cases of cholecystitis, need for preoperative ERCP, history of cholecystostomy, admission type, time from admission to surgical procedure, preoperative prediction of difficult cholecystectomy using the Nassar scale [20, 21], intraoperative findings (modified Nassar scale) [22], conversion rate, type of cholecystectomy (total or subtotal), use of drains, surgical time, procedure-related and hospitalization-related complications, length of hospital stay, need for reoperation, and mortality.

### Surgical procedure

Laparoscopic cholecystectomy was performed using the standard 4-port technique in the American position (1 umbilical, 1 subxiphoid, and 2 in the right hypochondrium). Dissection of the hepatocystic triangle was carried out until reaching the critical view of safety, always performing dissection above Rouviere’s sulcus and from lateral to medial. After achieving the critical view of safety, two proximal clips and one distal clip were placed separately on the cystic duct and cystic artery, followed by cutting between the clips and cysto-fundic dissection of the gallbladder. In cases where the critical view of safety could not be achieved, the surgeon discretely decided to perform the fundus first, subtotal cholecystectomy, or conversion to open. In none of the cases was intraoperative cholangiography or fluorescent cholangiography performed. It was also the surgeon’s discretionary decision to place a drain in the surgical bed [23, 24].

### Statistical analysis

For the demographic and clinical characterization of the patients, qualitative variables were statistically represented by their frequencies and percentages. For quantitative variables, measures of central tendency and measures of dispersion were presented depending on their distribution and nature (mean and standard deviation, or median and interquartile ranges). The normality of variables was assessed using the Shapiro‒Wilk test. Survival analysis was conducted using the Kaplan‒Meier survival curve method, and the comparisons between curves were made using the log-rank test for the ASA variable (ASA 1–2 versus ASA 3–4). Cox regression analysis was implemented to determine potential factors associated with mortality at 24 months. Proportional hazards assumptions and the influence of outliers were checked. A multivariate model was adjusted with variables that had a p-value less than 0.1 in the unadjusted analysis [25]. Additionally, the statistical power of the previously found estimates was determined. A p-value of less than 0.05 was considered statistically significant.

The analyses were conducted using STATA 17 statistical software and R language through RStudio interface software (2023).

## Results

A total of 144 patients were included in the study, of whom 37 (25.69%) died during the two-year follow-up period. The median age was 90 (IQR: 8.00) years, with a predominance of females (56.94%). Among the patients who died, a higher proportion had ASA 3–4 classification, cardiovascular disease, and a diagnosis of cholecystitis, with statistically significant differences observed. Other demographic, clinical, and surgical characteristics are described in Table 1.

**Table 1:** Demographic, clinical, and surgical characteristics according to survival at 24 months of follow-up Two-years survival status

	Total population (*n* = 144)	Alive at 2 years (*n* = 107)	Dead at 2 years (*n* = 37)	p-value
Age (y), median (IQR)	90.00 (8.00)	90.00 (8.00)	92.00 (6.50)	0.019*
Sex, n (%) Female Male	82 (56.94) 62 (43.05)	60 (56.07) 47 (43.92)	22 (59.45) 15 (40.54)	0.720
Body mass index (kg/m^2^), median (IQR)	24.20 (4.51)	24.29 (4.63)	24.00 (5.12)	0.651*
ASA classification, n (%) 1 2 3 4	2 (1.38) 62 (43.05) 74 (51.38) 6 (4.16)	2 (1.86) 54 (50.46) 47 (43.92) 4 (3.73)	0 (0.00) 8 (21.62) 27 (72.97) 2 (5.40)	**0.013**
Comorbidity, n (%) Diabetes mellitus Arterial hypertension Chronic obstructive pulmonary disease Chronic kidney disease Cardiovascular disease Liver disease	27 (18.75) 108 (75.00) 37 (25.69) 19 (13.19) 32 (22.22) 3 (2.08)	18 (16.82) 80 (74.76) 28 (26.16) 13 (12.14) 19 (17.75) 2 (1.86)	9 (24.32) 28 (75.67) 9 (24.32) 6 (16.21) 13 (35.13) 1 (2.70)	0.314 0.912 0.825 0.529 **0.028** 0.760
Charlson comorbidity index, median (IQR)	6.00 (2.00)	6.00 (2.00)	6.00 (1.00)	0.067*
Anticoagulants agents, n (%)	13 (9.02)	8 (7.47)	5 (13.51)	0.269
Antiplatelet agents, n (%)	32 (22.22)	23 (21.49)	9 (24.32)	0.721
Preoperative laboratories, median (IQR) Leukocytes (x10^3^) Hemoglobin (mg/dL) Bilirubins (mg/dL) Alkaline phosphatase (mg/dL) Aspartate aminotransferase (mg/dL) Alanine aminotransferase (mg/dL)	11.9 (3.37) 13.70 (2.65) 1.20 (1.78) 150.00 (148.50) 33.50 (75.50) 40.00 (106.75)	11.90 (6.90) 13.80 (2.60) 1.20 (1.73) 150.00 (140.00) 32.00 (102.00) 40.00 (106)	11.30 (6.45) 13.00 (3.60) 1.20 (1.94) 150.00 (183.50) 36.00 (60.00) 36.00 (106.50)	0.488* 0.010* 0.863* 0.443* 0.619* 0.389*
Bile duct diameter, (mm) median (IQR)	4.00 (4.00)	5.00 (4.00)	4.00 (2.50)	0.111*
Indication of surgical procedure, n (%) Biliary colic Pancreatitis Choledocholithiasis Acute cholecystitis	21 (14.58) 24 (16.66) 44 (30.55) 89 (61.80)	19 (17.75) 17 (11.88) 34 (31.77) 60 (56.07)	2 (5.40) 7 (18.91) 10 (27.02) 29 (78.37)	0.670 0.589 **0.016**
Classification of severity of cholecystitis, n (%) I II III	11 (7.63) 32 (22.22) 46 (31.94)	6 (56.07) 23 (21.49) 31 (28.97)	5 (13.51) 9 (24.32) 15 (40.54)	0.069
Preoperative ERCP, n (%) No Yes	92 (63.88) 52 (36.11)	67 (62.61) 40 (37.38)	25 (67.56) 12 (32.43)	0.589
Type of admission, n (%) Elective Delayed Emergency	9 (6.25) 126 (87.50) 9 (6.25)	8 (7.47) 95 (88.78) 4 (3.73)	1 (2.70) 31 (83.78) 5 (13.51)	0.071
History of cholecystostomy, n (%) No Yes	139 (96.52) 5 (3.47)	105 (98.13) 2 (1.86)	34 (91.89) 3 (8.10)	0.074
Time from admission to surgical procedure (days), median (IQR)	6.00 (6.50)	6.00 (6.00)	8.00 (6.50)	0.107*
Preoperative risk scale for difficult laparoscopic cholecystectomy, median (IQR)	9.00 (5.00)	8.00 (6.00)		0.013*
Intraoperative findings according to Nassar scale 1 2 3 4 5	36 (25.00) 33 (22.91) 23 (15.97) 24 (16.66) 28 (19.44)	28 (26.16) 24 (22.42) 17 (15.88) 19 (17.75) 19 (17.75)	8 (21.62) 9 (24.32) 6 (16.21) 5 (13.51) 9 ((24.32)	0.882

The p values were obtained from the Chi-square test

*The p values were obtained from the Mann–Whitney test

Bold values indicate statistically signifcant p values (*p* < 0.05)

Regarding surgical outcomes, it was observed that patients who died during the follow-up had a higher proportion of subtotal cholecystectomy, conversion to open surgery, reintervention, and overall complications. However, statistically significant differences were not observed in these variables between the analyzed groups **(**Table 2**)**.

**Table 2:** Surgical Outcomes According to Survival at 24 Months of Follow-up Two-years survival status

	Total population (*n* = 144)	Alive at 2 years (*n* = 107)	Mortality at 2 years (*n* = 37)	p-value
Conversion rate, n (%)	19 (13.19)	12 (11.21)	7 (18.91)	0.233
Type of cholecystectomy, n (%) Total Subtotal	126 (87.50) 18 (12.50)	97 (90.65) 10 (9.34)	29 (78.37) 8 (21.62)	0.052
Surgical time (minutes), median (IQR)	90.00 (48.75)	85.00 (40.50)	90.00 (42.00)	0.229*
Drain use, n (%) No Yes	108 (75.00) 36 (25.00)	83 (77.57) 24 (22.42)	25 (67.56) 12 (32.43)	0.226
Length hospital stay (days), median (IQR)	10.00 (7.00)	10.00 (8.00)	11.00 (7.00)	0.077*
Complications, n (%) Bile duct injury Bleeding Intestinal injury Surgical site infection Acute myocardial infarction perioperative Pulmonary embolism perioperative Health care-associated pneumonia Health care-associated urinary tract infection Pleural efussion	8 (5.55) 9 (6.25) 1 (0.69) 6 (4.16) 2 (1.38) 3 (2.08) 2 (1.38) 6 (4.16) 7 (4.86)	5 (4.67) 7 (6.54) 1 (0.93) 3 (2.80) 0 (0.00) 1 (0.93) 2 (1.86) 3 (2.80) 4 (3.73)	3 (8.10) 2 (5.40) 0 (0.00) 3 (8.10) 2 (5.40) 2 (5.40) 0 (0.00) 3 (8.10) 3 (8.10)	0.432 0.806 0.555 0.164 **0.015** 0.101 0.402 0.164 0.287
Reintervention, n (%) No Yes	131 (90.97) 13 (9.02)	100 (93.45) 7 (6.54)	31 (83.78) 6 (16.21)	0.077

The p values were obtained from the chi-square test

*The p values were obtained from the Mann–Whitney test

Bold values indicate statistically signifcant p values (*p* < 0.05)

**Fig. 1:**
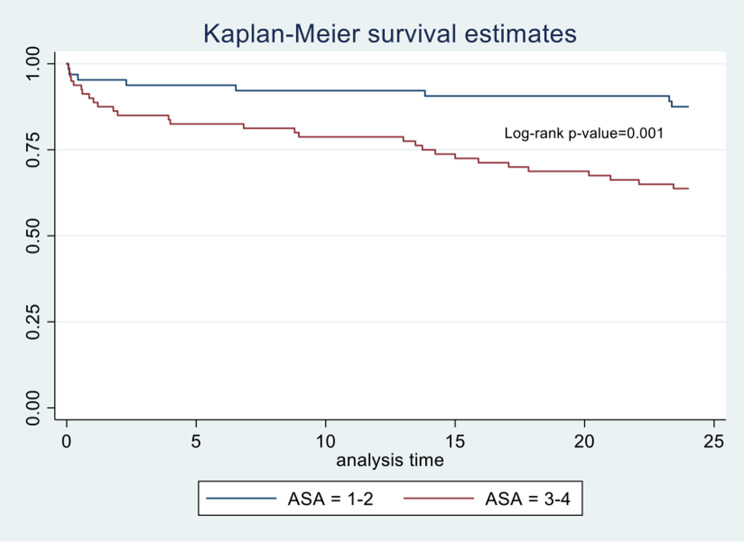
Kaplan‒Meier curve for time to death depending on ASA classification

Survival curves were compared for different ASA groups and statistically significant differences were determined through the log-rank test, showing a higher proportion of survivors at two years in patients who were ASA 1–2 at 87.50%, compared to ASA 3–4 at 63.75% (*p* = 0.001) (Fig. 1).

A Cox regression analysis was performed to determine factors associated with mortality. The proportional hazards assumption and the influence of outliers were verified through graphical methods. An unadjusted analysis was conducted with variables that met the proportional hazards assumption, and those that showed statistical significance in the unadjusted analysis were included in the multivariate model. The ASA variable was identified as a statistically significant factor associated with mortality, revealing a higher risk (HR: 2.71, CI95%:1.20–6.14) in the ASA 3–4 group than in the ASA 1–2 group. Although the cholecystitis variable did not show a statistically significant association, patients with this clinical condition had twice the risk compared to those without cholecystitis **(**Table 3**)**.

**Table 3:** Cox regression analysis of long-term mortality in patients ≥ 80 years treated with laparoscopic cholecystectomy

	Crude HR (CI95%)	Multivariable adjusted HR (CI95%)*	Multivariable adjusted HR (CI95%)**
Sex	0.88 (0.45–1.70)		
ASA classification	3.32 (1.15–7.28)	2.71 (1.20–6.14)	3.00 (1.36–6.62)
Charlson comorbidity index	1.19 (0.96–1.48)		
Cardiovascular disease	2.17 (1.10–4.26)	1.47 (0.72–2.98)	
Cholecystitis	2.47 (1.13–5.41)	2.03 (0.91–4.49)	2.12 (0.91–4.67)
ERCP	0.85 (0.43–1.70)		

*Multivariable model includes ASA classification, cardiovascular disease and cholecystitis

**Multivariable model includes ASA classification, cholecystitis

Reference value: Sex = Female, ASA classification: 1–2, Charlson comorbidity index = 0, Cardiovascular disease = No, Cholecystitis = No, ERCP = No

The statistical package powerSurvEpi [26] was used, which allows for the determination of power for a Cox regression with two dichotomous covariates. The HR value obtained for the ASA variable, the proportion of deceased patients, the proportion of patients with ASA 3–4, and the total number of patients were used in this analysis. A low correlation of 0.2 between the ASA score and cholecystitis was assumed. A statistical power of 90% was obtained.

## Discussion

Laparoscopic cholecystectomy is the gold standard for the treatment of benign biliary disease, with low rates of morbidity and mortality in the general population [27]. However, in older adults, especially those in their eighties and nineties, surgical outcomes are worse, with an odds ratio (OR) for mortality in patients over 80 years of age of 10.20 (95% CI 4.97–20.92) [6] and a 6.8% mortality rate at 30 days for those over 90 years old [15]. Long-term mortality after cholecystectomy has been reported in 2% of patients, but when evaluating long-term mortality in patients over 80 years old, it can reach up to 32% [1, 28].

Considering the higher risk of morbidity and mortality in patients over 80 years of age, both in terms of postoperative complications at 30 days and long-term outcomes, it is necessary to evaluate different treatment alternatives for this patient group to improve outcomes. Among therapeutic alternatives are cholecystostomy, antibiotics, and/or ERCP. Conservative management has been evaluated with a long-term recurrence rate of 39.8% [10]. In another study that evaluated conservative management, a readmission rate of 39.2% was observed in cases where cholecystostomy was performed and 38.1% in cases where only conservative management without cholecystostomy was implemented [29]. Cholecystectomy has also been compared to cholecystostomy in high-risk patients (defined as patients with APACHE II ≥ 7), with no evidence of differences in mortality but a higher proportion of complications in patients who underwent cholecystostomy [30]. In another study in patients aged ≥ 90 years, cholecystectomy was compared to cholecystostomy in patients with cholecystitis, finding a higher proportion of complications and 30-day mortality in patients who underwent laparoscopic cholecystectomy, although without statistically significant differences [31]. Wiggins et al. published a retrospective study based on a national administrative database of all patients over 80 years of age with AC, including 47,500 patients. They found a one-year mortality rate of 27.1% with conservative management, 35.0% with cholecystostomy, and 20.8% with cholecystectomy. These results favor the performance of laparoscopic cholecystectomy in patients over 80 years of age [32].

In our results, we found a cumulative mortality rate at 2 years of follow-up of 25.69%, of which 7.63% died in the first 30 days after the operation. This implies, as previously shown, a much higher mortality rate in this population than in the general population, both in the short and long term. However, when comparing these proportions with the proportions of disease recurrence, the proportions of mortality are lower than the proportions of disease recurrence. Therefore, one might consider it better to perform the surgical procedure (laparoscopic cholecystectomy) because if the disease recurs, additional treatment will be needed, which could be associated with complications.

The length of hospital stay in patients who died during the 2-year follow-up was higher compared to patients who survived, although without a statistically significant difference. This may be related to a higher morbidity in this group of patients. It is also necessary to consider that hospitalization is influenced by the economic status of each country, hospitalization practices, insurance, and territorial distances between residence and hospital, which can influence the length of hospital stay [33].

Subsequently, we conducted a Cox regression in which we found ASA 3–4 classification as a factor associated with a higher proportion of mortality during follow-up (HR: 3.00) and a statistically significant difference between the Kaplan‒Meier curves of patients with ASA 1–2 and ASA 3–4, with an accumulated proportion of 36.25% at 2 years of follow-up, which is similar to the reported recurrence rate.

In light of these results, we could consider conservative treatment in patients with benign biliary disease who have ASA classification 3–4 because their 2-year mortality rate is similar to disease recurrence, and disease recurrence is a more favorable outcome than mortality. However, it is important to note that if disease recurrence occurs, it will likely require additional treatment, which may be surgical with the subsequent risk of complications. This is not very different from what is already recommended in some guidelines for the management of acute cholecystitis, where patients with anesthesia contraindication should not undergo laparoscopic cholecystectomy but rather receive antibiotic management, and if that fails, cholecystostomy [34].

This study presents some limitations. First, this is a retrospective study based on the data recollected previously by the surgery department, so some relevant information was unavailable. Second, data about the functional, cognitive, frailty, and nutritional status was not recollected and hence considered in the analysis. These variables could modify the outcomes in older adults who need a surgical procedure. Third, the cause of death was not considered, so we only performed analysis by mortality for all causes.

Further studies are needed in this special population of patients, which is steadily increasing, to provide recommendations on the best therapeutic approach [35, 36]. Currently, there is still limited evidence and consensus regarding the optimal therapeutic approach in this patient group [34, 37].

## Conclusions and implications

Octogenarian and nonagenarian patients with ASA 1–2 classification may benefit from laparoscopic cholecystectomy for the management of benign biliary disease. Conversely, ASA 3–4 patients may benefit from conservative management, such as cholecystostomy or antibiotics, due to their higher risk of mortality at 2 years and a lower likelihood of disease recurrence. However, the decision of performing a surgery procedure in older adults, especially with very older, have to consider the comprehensive geriatric assessment.

## Data Availability

The data used in the present study are available upon request to the corresponding author.
